# P-1816. Stepwise Implementation of an Antimicrobial Stewardship Prospective Audit and Feedback Intervention at a Large Academic Center in Canada

**DOI:** 10.1093/ofid/ofae631.1979

**Published:** 2025-01-29

**Authors:** Teagan Zeggil, Carlos Cervera, Stephanie W Smith, Dima Kabbani, Serena Bains, Cecilia Lau, Karen G Fong, Jackson J Stewart, Karen Doucette, Justin Chen

**Affiliations:** Alberta Health Services, Edmonton, Alberta, Canada; University of Alberta, Edmonton, Alberta, Canada; University of Alberta, Edmonton, Alberta, Canada; University of Alberta, Edmonton, Alberta, Canada; Alberta Health Services, Edmonton, Alberta, Canada; Alberta Health Services, Edmonton, Alberta, Canada; Alberta Health Services, Edmonton, Alberta, Canada; Alberta Health Services, Edmonton, Alberta, Canada; University of Alberta, Edmonton, Alberta, Canada; University of Alberta, Edmonton, Alberta, Canada

## Abstract

**Background:**

Effective implementation strategies for establishing antimicrobial stewardship (AMS) program inpatient interventions are not well described. We describe a methodology for prospective audit and feedback (PAF) implementation at a large Canadian hospital and report its outcomes.

**Methods:**

A PAF intervention targeting restricted antibiotics (carbapenems, daptomycin, linezolid, and tigecycline) was launched in March 2018. Stepwise implementation by inpatient programs was prioritized by collaboration readiness as immediate site-wide implementation was deemed unfeasible. New prescriptions were assessed by the antimicrobial stewardship team. Prescription, prescriber, intervention type, and acceptance data were collected through March 2024.

**Results:**

Over a 2-year period, site wide stepwise implementation occurred in 16 discrete phases. Over the study period, 5185 prescriptions were evaluated; 3789 (73%) were empiric, and 1396 (27%) culture directed from which 3604 (70%) were optimally prescribed (evaluated by agent, regimen, and duration). Of empiric prescriptions, 636 (17%) were not guideline-concordant by indication, 1102 (29%) did not follow guidelines but was reasonable by expert opinion, and 2051 (54%) were guideline-concordant.

ASP recommendations included: 3604 no change (70%), 864 change agent (17%), 535 change regimen (10%), 172 discontinue antibiotic (3%), and 10 other (0.2%). The recommendation acceptance rate was 91% (1581 cases evaluable).

Infectious diseases (ID) were involved in 1466 (28%) prescriptions. With ID involvement, higher guideline-concordant empiric prescribing (64% vs 51%, p< 0.001) and regimen appropriateness (85% vs 65%, p< 0.001) was observed. ASP suggested no changes more often with ID involvement (84% versus 62%, p< 0.001).

**Conclusion:**

Stepwise implementation allowed evolution and implementation of ASP processes and workflow. 30% of prescriptions audited resulted in intervention, suggesting a possible role for further education, guidelines, or restriction. ID involvement was associated with more optimal prescribing. A stepwise implementation of an antimicrobial stewardship PAF intervention is a viable strategy.

**Disclosures:**

**All Authors**: No reported disclosures

